# IL-10 and Lymphotoxin-α Expression Profiles within Marginal Zone-Like B-Cell Populations Are Associated with Control of HIV-1 Disease Progression

**DOI:** 10.1371/journal.pone.0101949

**Published:** 2014-07-08

**Authors:** Josiane Chagnon-Choquet, Julie Fontaine, Johanne Poudrier, Michel Roger, for the Montreal Primary HIV Infection and Slow Progressor Study Groups

**Affiliations:** 1 Laboratoire d'Immunogénétique, Centre de Recherche du Centre Hospitalier de l'Université de Montréal (CRCHUM), Montréal, Quebec, Canada; 2 Département de Microbiologie, Infectiologie et Immunologie de l'Université de Montréal, Montréal, Quebec, Canada; Vita-Salute San Raffaele University School of Medicine, Italy

## Abstract

Understanding how the immune system facilitates or controls HIV-1 disease progression has important implications for the design of effective interventions. We report that although B-cell dysregulations associated with HIV-1 disease progression are accompanied by an overall decrease in the percentage of total blood B-cells, we observe an increase in relative frequencies of cells presenting characteristics of both transitional immature and first-line marginal zone (MZ) B-cell populations, we designated as precursor MZ-like B-cells. B-cells with similar attributes have been associated with IL-10 expression and “regulatory” potential. As such, the relative frequencies of precursor MZ-like B-cells expressing IL-10 are increased in the blood of viremic HIV-1-infected individuals when compared to HIV-negative subjects. Importantly, in aviremic HIV-1 Elite-Controllers (EC), we found unaltered relative percentages of precursor MZ-like B-cells which presented normal IL-10 expression patterns. Furthermore, EC had increased relative frequencies of blood MZ-like B-cells expressing LT-α. Thus in contrast to viremic HIV-1-infected individuals, EC present MZ-like B-cell populations which IL-10 and LT-α expression profiles may favour homeostasis of immune responses and lymphoid microenvironments.

## Introduction

It is well known that the contribution of the B-cell compartment to effective viral control is impeded in the vast majority of HIV-1-infected individuals. Indeed, B-cell dysregulations are observed early, persist throughout the course of infection, and are not fully restored by therapy. These B-cell alterations favour the overall inflammatory burden and often lead to autoimmune manifestations and malignancies [1]–[2].

We have previously shown that HIV-transgenic (Tg)-mice, which develop a negative factor (Nef)-dependant AIDS-like disease [3], present B-cell dysregulations that are similar to those reported for HIV-infected individuals [4]. Strikingly, these animals present an enlarged splenic marginal zone (MZ), in which accumulated myeloid dendritic cells (mDCs) likely contribute to MZ expansion, polyclonal B-cell activation and breakage of tolerance through delivery of excessive signals such as B lymphocyte stimulator (BLyS/BAFF) [4]–[5]. A similar B-cell profile was reported for BLyS/BAFF-Tg [6] and autoimmune-regulatory-(AIRE)-deficient mice, in which BLyS/BAFF is elevated in serum and over-expressed by DCs [7]–[8]. Accordingly, DCs play a pivotal role in regulating B-cell development, activation and survival mainly through production of growth factors such as BLyS/BAFF [9]–[11], known to highly influence the transitional immature (TI) and MZ B-cell pools [12]–[13].

MZ B-cells constitute early first-line defence against invading pathogens. In humans, they likely constitute a heterogeneous niche that is not restricted to the spleen, as they have been found in blood, lymphoid organs and mucosal-associated structures [12]–[13]. Reminiscent of what had been observed in HIV-Tg mice [4], we found that B-cell dysregulations in HIV-1-infected rapid and classic progressors were accompanied by increased relative frequencies within total blood B-cells of a population presenting features shared by both TI and MZ B-cells, which we designated “precursor” MZ-like B-cells. Concomitantly, these individuals presented increased BLyS/BAFF levels in blood and on surface of blood mDCs [14]. In contrast, relative frequencies of precursor MZ-like B-cells as well as BLyS/BAFF expression status were unaltered in HIV-1 Elite-Controllers (EC) [14]. Instead, decreased frequencies of more “mature” MZ-like B-cells were observed in the blood B-cell compartment of EC.

B-cells are involved in regulating the development, proliferation and maintenance of CD4^+^ T-cell populations, through both contact and/or cytokine mediated effector and regulatory functions [15]. Regulatory “Breg” potential has not yet been attributed to a particular B-cell population and relies rather on IL-10 expression/production and function. Both precursor and mature B-cells with MZ attributes as well as TI and memory populations have been ascribed such Breg potential [16]–[17]. Recently, increased percentages of Breg producing IL-10 were observed in chronically HIV-infected subjects [18]. This has prompted us to assess whether IL-10 expression profiles by precursor and mature MZ-like as well as TI and memory B-cell populations are modulated during HIV-1 infection. In addition, the fact that high levels of Lymphotoxin (LT)-α have been associated with autoimmune and inflammatory contexts [19], and that increased LT-α to IL-10 B-cell expression ratios have been observed in patients with multiple sclerosis [20], prompted us to also assess B-cell LT-α expression profiles. We show increased frequencies of total B-cells expressing IL-10 in the blood of HIV-infected rapid and classic progressors as compared to those observed in HIV-uninfected donors. The most significant increase in cells expressing IL-10 is within the precursor MZ-like B-cell population. Furthermore, in contrast to viremic HIV-1-infected individuals, EC present MZ-like B-cell populations which IL-10 and LT-α expression profiles may favour homeostasis of immune responses and lymphoid microenvironments.

## Methods

### Ethics Statement

Written informed consent was obtained from all subjects, and the research conformed to ethical guidelines and was approved by the Ethics Review board of the Centre de Recherche du CHUM. The reference number for the project is: SL 05.028.

### Subjects

Thirty male HIV-1-infected subjects were selected from the Montreal Primary HIV Infection (PHI) cohort: 13 were classified as rapid and 17 as classic progressors. The date of infection was estimated using criteria established by the Acute HIV Infection and Early Disease Research Program (NIAID, Bethesda, MD). Rapid progressors had blood CD4^+^ T-cell counts below 250 cells/mm^3^ within 2 years of infection. Blood samples were taken in acute (0–3 months) and/or early (5–8 months) phases of infection, and 3–6 and 9–12 months after initiation of antiretroviral therapy (ART). Classic progressors were ART-naive individuals whose blood CD4^+^ T-cell counts remained above 500 cells/mm^3^ for the 2 year follow-up. Blood samples were obtained in the acute, early and chronic (24 months) phases of infection. Moreover, blood samples from 13 slow progressors were obtained from the Slow Progressors cohort, where patients were infected for 8 years or more with blood CD4^+^ T-cell counts above 500 cells/mm^3^ and presented low (7 viremic patients) to undetectable (6 aviremic patients/EC) viral loads in absence of ART. Blood samples from 20 age- and sex-matched HIV-negative healthy volunteers were also obtained. HIV viral loads were determined in plasma using Versant HIV-1 RNA 3.0 Assay (Siemens Medical Solutions Diagnostics, Tarrytown, NY). Blood CD4^+^ T-cell counts were determined as reported [21]. None of the subjects had syphilis, hepatitis B or C.

### Evaluation of plasma IL-10 and LT-α concentrations

Plasma levels of IL–10 were determined using the Cytometric Bead Array Human Inflammation Kit (BD-Biosciences, San Jose, CA, USA) according to the manufacturer's instructions. Data were acquired on a FACSAria and analysed with the FCAP software (BD-Biosciences). Plasma levels of LT-α were measured with the Milliplex Map magnetic bead kit (Millipore, Billerica, MA, USA) according to the manufacturer's protocol using the Luminex 200 Total System (Luminex Corporation, Austin, TX, USA).

### Evaluation of blood B-cell populations by flow-cytometry

Peripheral blood mononuclear cells (PBMCs) were isolated on Ficoll gradient, resuspended in heat-inactivated fetal bovine serum (hi-FBS) containing 10% dimethyl sulfoxyde and stored in liquid nitrogen. One million PBMCs per sample were used for staining. Live/dead exclusion was performed using Aqua-LIVE/DEAD Fixable Stain (Invitrogen Life technologies, Eugene, OR, USA). Non-specific binding sites were blocked using fluorescence-activated cell sorting (FACS) buffer (1× PBS, 2% hi-FBS, and 0.1% sodium azide) supplemented with 20% hi-FBS and 10 µg mouse IgG (Sigma-Aldrich, St-Louis, MO, USA). The following conjugated mouse anti-human monoclonal antibodies were used: PacificBlue-anti-CD19, APC-Cy7-anti-CD10 (BioLegend, San Diego, CA, USA), AlexaFluor700-anti-CD27, FITC-anti-IgM, PE-anti-CD21 (BD-Biosciences), PerCP-eFluor710-anti-CD1c (eBioscience, San Diego, CA, USA), APC-anti-LT-α (LifeSpan BioSciences, Seattle, WA, USA), and rat anti-human PE-Cy7-anti-IL-10 (BioLegend). Intracellular labelling was performed using the Cytofix/Cytoperm Fixation/Permeabilization kit and perm/wash buffer (BD-Biosciences). Intracellular non-specific binding sites were blocked using perm/wash buffer containing 20% hi-FBS, 50% rat serum and 20 µg mouse IgG. Cells were kept at 4°C in 1.25% paraformaldehyde for 18 hours prior to analysis. Data acquisition of 10^5^ events per sample was performed with LSRFortessa (BD-Biosciences), and analysis was done with FlowJo7.6.3 software (TreeStar, Ashland, OR, USA). All stainings were compared to that of fluorescence minus one (FMO) values and isotype controls (see Figure S1 for details). Anti-mouse Ig(κ) Compbeads and CS&T Beads (BD-Biosciences) were used to optimize fluorescence compensation settings and calibrate the LSRFortessa, respectively.

### Statistical analyses

The statistical significance of differences between groups was assessed with Fisher exact test for categorical variables and unpaired Student t test or one-way analysis of variance when continuous variables were normally distributed or with Mann-Whitney U test otherwise. Wilcoxon signed rank test was used for pairwise comparisons of different phases of infection within each group. Spearman rank test was used to determine correlations between continuous variables. Outliers' samples were withdrawn from statistical analyses when the values were greater than the mean plus three times standard deviation. Analyses were performed using GraphPad Prism 5.03 for Windows (GraphPad Software Inc, La Jolla, CA, USA).

## Results

### Socio-demographic and clinical characteristics of HIV-1-infected individuals

The socio-demographic and clinical characteristics of the HIV-1-infected individuals are shown in Table 1, and the longitudinal assessment of blood CD4^+^ T-cell counts and viral loads are depicted in Figure S2A-B. There were no significant correlations between blood CD4^+^ T-cell counts or viral loads and B-cell populations, LT-α and IL-10 plasma levels studied here either within groups or among all subjects during early or chronic infection (data not shown).

**Table 1:** Sociodemographic and clinical characteristics of HIV-infected individuals.

	Rapid progressors	Classic progressors	Viremic slow progressors	Aviremic slow progressors	*P*
**Characteristics**	(n = 13)	(n = 17)	(n = 7)	(n = 6)	
Age at first visit (years)	34±7	38±8	46±9	44±9	0.005^a^
Sex (male/female)	12/1	17/0	7/0	3/3	0.005
Race (white/other)	12/1	16/1	6/1	5/1	NS
**CD4+ T cells/mm^3^**					
Acute phase	533±140	781±213	na	na	0.002
Early phase	446±40	714±46	na	na	0.0008
Chronic phase	443±193	629±244	562±122	888±122	0.011^b^
Nadir	254±118	432±140	506±129	506±175	0.0005^c^
**Viremia, ×10^3^ copies/ml** d					
Acute phase	366±705	76±126	na	na	NS
Early phase	121±186	71±190	na	na	NS
Chronic phase	7.65±15.3	37.7±62.3	3.01±1.86	<0.05^d^	0.003^e^
Peak	570±808	202±236	8.5±6.5	0.065±0.027	0.0001^f^

Age, CD4 and viremia are expressed as mean ±SD. Sex and race were compared using Fisher exact test. Pairwise comparisons of CD4 and viremia for early phases were performed using unpaired Student's *t* tests. Comparisons among all groups (age at first visit, CD4, viremia in the chronic phase, nadir CD4) were performed with the one-way analysis of variance test. n, numbers; NS, not significant; na, not available.

a P = 0.004 and 0.050 for the comparison of age between rapid and viremic slow progressors, and classic and viremic slow progressors, respectively, as determined by the Mann-Whitney test.

b P = 0.008 for the comparison of CD4+ T cells/mm^3^ in chronic phase between rapid progressors and aviremic slow progressors, as determined by the Mann-Whitney test.

c P = 0.0008 and 0.001 and 0.020 nadir CD4 for the comparison between rapid and classic progressors, rapid and viremic slow progressors, and rapid and aviremic slow progressors, respectively, as determined by the Mann-Whitney test.

d Fifty copies/ml corresponds to the detection threshold of the viral load test.

e P = 0.002 and 0.002 for the comparison of viremia in chronic phase between classic progressors and aviremic slow progressors, and viremic and aviremic slow progressors, respectively, as determined by the Mann-Whitney test.

f P = 0.006, 0.0007, 0.0005, 0.0004 and 0.001 for the comparison of peak viremia between rapid progressors and viremic slow progressors, rapid progressors and aviremic slow progressors, classic progressors and viremic slow progressors, classic progressors and aviremic slow progressors, and viremic and aviremic slow progressors, respectively, as determined by the Mann-Whitney test.

### Longitudinal monitoring of blood B-cell populations in HIV-1-infected individuals with different rates of disease progression

**Figure 1:**
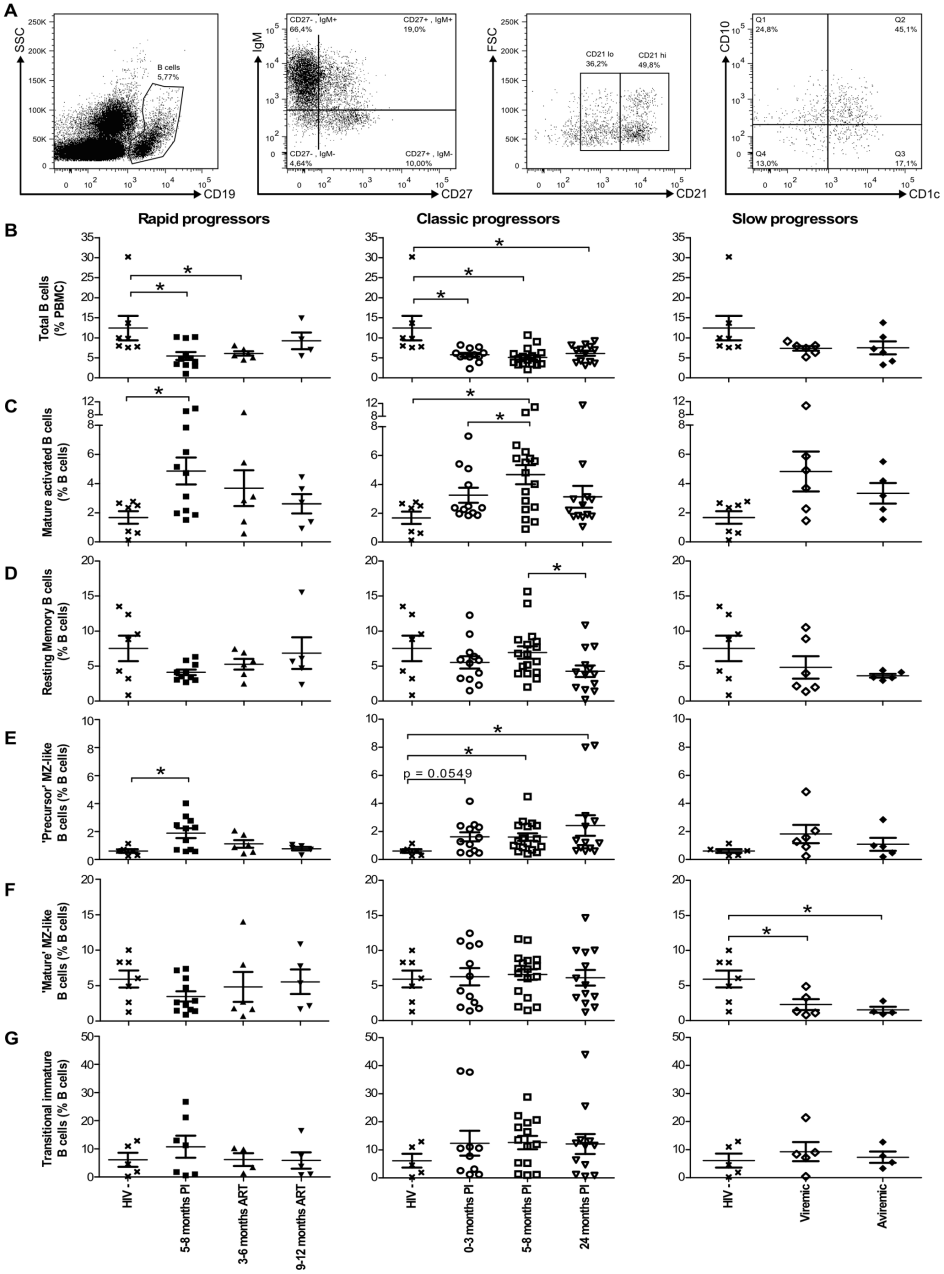
Longitudinal analysis of blood B-cell populations of HIV-1 infected individuals. (A) Representative plot showing gating strategy on live PBMCs. Total CD19+ B-cells were selected based on expression of CD27 and/or IgM, and levels of CD21. CD1c and CD10 expression were used for further characterisation of blood MZ and TI B-cell populations respectively, as reported [13]. Quadrants were set based on the expression values obtained with fluorescence minus one (FMO) and isotype controls. Mature activated B-cells are defined as CD19+CD27+IgM-CD21loCD1c-CD10-, resting switched memory B-cells are CD19+CD27+IgM-CD21hiCD10-, precursor marginal-zone (MZ)-like B-cells are CD19+CD27+IgM+CD21loCD1c+ CD10+, mature MZ-like B-cells are CD19+CD27+IgM+CD21hiCD1c+CD10- and transitional immature (TI) B-cells are CD19+CD27-IgM+CD21hiCD1c-CD10+. The graphs present (B) percentages of total B-cells (mean events gated: 9320±1750), and the relative frequencies of (C) mature activated (mean events gated: 360±67), (D) resting switched memory (mean events gated: 632±301), (E) precursor MZ-like (mean events gated: 145±36), (F) mature MZ-like (mean events gated: 327±233) and (G) TI (mean events gated: 944±174) B-cell populations in the blood of rapid progressors (left panels; 5–8 months PI (n = 11), 3–6 months ART (n = 6) and 9–12 months ART (n = 5)), classic progressors (middle panels; 0–3 months PI (n = 12), 5–8 months PI (n = 17), and 24 months PI (n = 13)), and viremic and aviremic slow progressors (EC) (right panels; viremic (n = 6); aviremic (n = 5)). The same values for HIV-negative donors in the left, middle and right panels are used as a control group (n = 7). Cell population frequencies were compared using the Wilcoxon signed rank test and the Mann-Whitney U test for pairwise comparisons of different phases of infection within each group and between the study groups, respectively. Data shown are mean ±SEM. * p<0.05. PI, post-infection; ART, antiretroviral therapy.

We have previously reported that the frequencies of blood precursor and mature MZ-like B-cell populations are different in the context of HIV disease control or progression [14]. To further the characterization of these B-cells, we have adapted our staining cocktail for the detection of intracellular expression of IL-10 and LT-α (see below). The analyses of B-cell populations using this modified staining strategy confirm previous observations and are depicted in Figure 1. The mean percentages of total live blood CD19^+^ B-cells in all HIV-1-infected individuals were lower throughout the course of infection than those observed in HIV-negative donors, reaching statistical significance in both rapid and classic progressors (Figure 1B). Upon analysis for variations in the relative frequencies of different B-cell populations, we observed that the mean relative percentages of mature activated (CD19^+^CD27^+^IgM^−^CD21^l^°CD10^−^) and precursor MZ-like B-cells (CD19^+^CD27^+^IgM^hi^CD21^l^°CD1c^+^CD10^+^), were significantly elevated in rapid and classic progressors (Figure 1C, E). There was a trend for increased relative frequencies of TI B-cells (CD19^+^CD27^−^IgM^+^CD21^+^CD10^+^) in chronically HIV-1-infected classic progressors (Figure 1G). In contrast, the relative frequency of the more mature MZ-like B-cell population (CD19^+^CD27^+^IgM^hi^CD21^hi^CD1c^+^CD10^−^) within total blood B-cells remained unaltered in both rapid and classic progressors (Figure 1F, left and middle panels), whereas the percentages were significantly decreased in both viremic and aviremic slow progressors, the latter also referred herein as EC (Figure 1F, right panel). The relative frequencies of resting switched memory B-cells (CD19^+^CD27^+^IgM^−^CD21^hi^CD10^−^) were decreased in chronically infected classic progressors (Figure 1D, middle panel). Naïve resting B-cells (CD19^+^CD27^−^IgM^+^CD21^hi^CD10^−^), were slightly lower in all HIV-1-infected individuals (data not shown).

### Longitudinal monitoring of IL-10 expression by blood B-cell populations in HIV-1-infected individuals with different rates of disease progression

**Figure 2:**
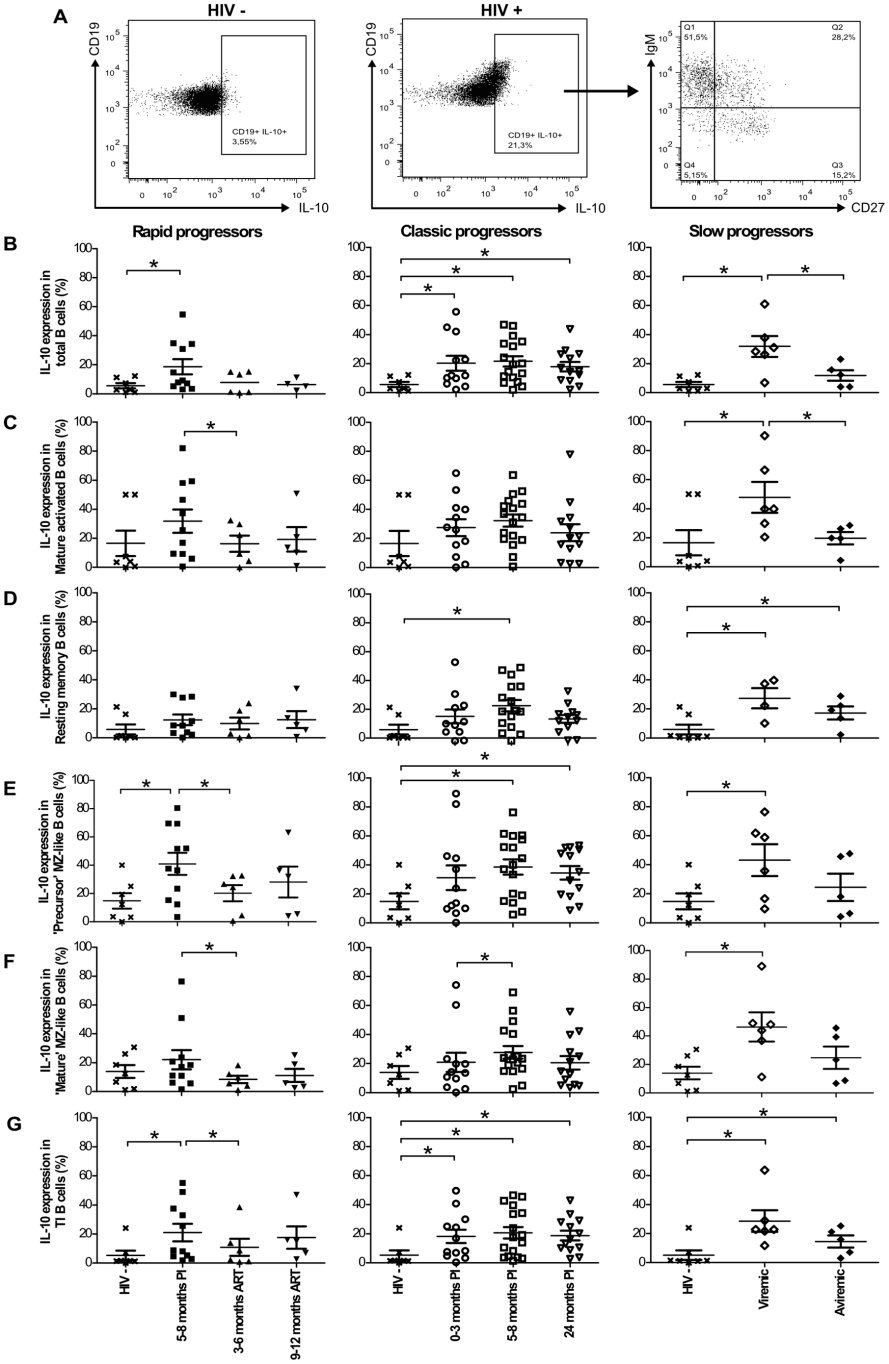
IL-10 expression by blood B-cell populations. (A) Representative plot showing gating strategy on live total CD19+ B-cells from HIV- and HIV+ donors, expressing IL-10. Frequencies of cells expressing IL-10 within (B) total, (C) mature activated, (D) resting switched memory, (E) precursor marginal zone (MZ)-like, (F) mature MZ-like and (G) transitional immature (TI) B-cell populations in the blood of rapid progressors (left panels; 5–8 months PI (n = 11), 3–6 months ART (n = 6) and 9–12 months ART (n = 5)), classic progressors (middle panels; 0–3 months PI (n = 12), 5–8 months PI (n = 17), and 24 months PI (n = 13)), and viremic and aviremic slow progressors (EC) (right panels; viremic (n = 6); aviremic (n = 5)). The same values for HIV-negative donors in the left, middle and right panels are used as a control group (n = 7). Data are expressed as percentages of IL-10 expression within each B-cell population. Cell population frequencies were compared using the Wilcoxon signed rank test and the Mann-Whitney U test for pairwise comparisons of different phases of infection within each group and between the study groups, respectively. Data shown are mean ±SEM. * p<0.05. PI, postinfection; ART, antiretroviral therapy.

Blood B-cell populations presented in Figure 1 were assessed *ex vivo* for intracellular IL-10 expression based on the same strategy of flow-cytometry analysis depicted in Figure 1A. Figure 2A shows a representative profile of IL-10 expression on total live B-cells from HIV- and HIV+ donors, and distribution within IgM and CD27 quadrants of expression. Note that the majority of IL-10 expressing B-cells are IgM^+^ (Figure 2A). Because IL-10 expression might be influenced by the fluctuations in B-cell population frequencies shown in Figure 1, data are expressed as percentages of IL-10 expressing cells within each B-cell population. Frequencies of total B-cells expressing IL-10 in all viremic HIV-1-infected individuals were significantly higher than those observed in HIV-negative donors (Figure 2B). The percentages of total B-cells expressing IL-10 were restored to normal levels following therapy in rapid progressors (Figure 2B, left panel). Similar patterns of IL-10 expression were observed for precursor MZ-like and TI B-cell populations in all viremic HIV-1-infected individuals (Figure 2E, G), as well as for mature activated and resting switched memory B-cells in viremic slow progressors (Figure 2C-D). Relative frequencies of resting switched memory B-cells expressing IL-10 were increased in classic progressors during the early phase of infection (Figure 2D, middle panel), whereas those of mature MZ-like B-cells expressing IL-10 were elevated in viremic slow progressors (Figure 2F, right panel). Importantly, percentages of IL-10 expression in all B-cell populations of aviremic slow progressors/EC were similar to those observed in HIV-negative donors, except for resting switched memory and TI B-cells, in which the relative frequencies of IL-10 expressing cells were significantly increased (Figure 2D, G, right panels). The frequencies of naïve resting B-cells expressing IL-10 remained unaltered in all HIV-infected individuals (data not shown). We did not detect changes in IL-10 expression levels following analysis of geometric mean fluorescence intensities (geoMFI) (data not shown). Figure S3 shows a recapitulative analysis of B-cell IL-10 expression profiles. The heterogeneity of CD19 expression may be explained by the presence of blasts or other B-cell populations that were not specifically analyzed in the present study. The contribution to IL-10 expression from B-cell populations not analyzed herein will require further investigation.

**Figure 3:**
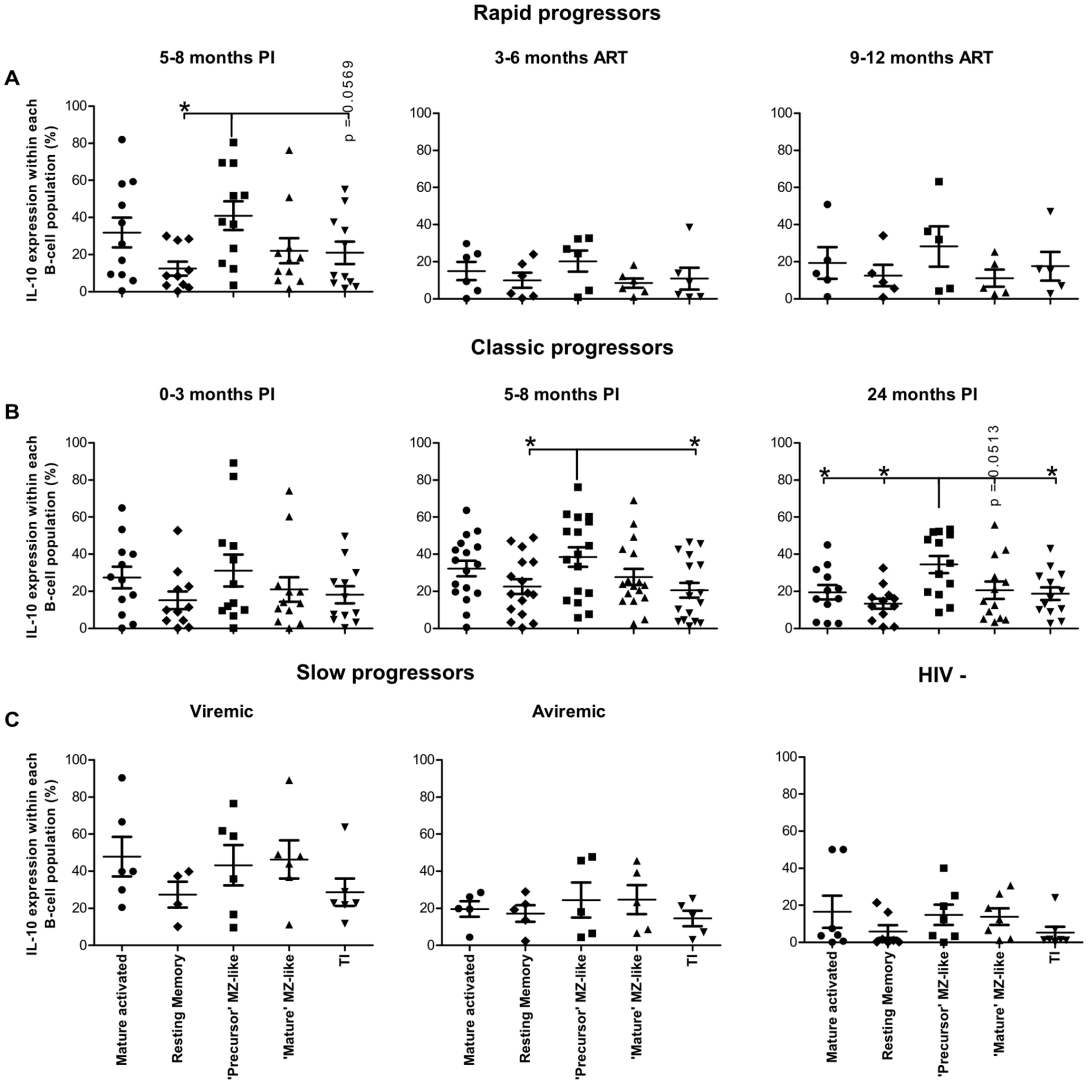
Percentage of IL-10 expressing cells for each B-cell population, within each patient group. Percentage of IL-10 expressing cells within mature activated, resting switched memory, precursor marginal zone (MZ)-like, mature MZ-like and transitional immature (TI) B-cells within (A) rapid progressors (5–8 months PI (n = 11), 3–6 months ART (n = 6) and 9–12 months ART (n = 5), (B) classic progressors (0–3 months PI (n = 12), 5–8 months PI (n = 17), and 24 months PI (n = 13)), (C) slow progressors (viremic (n = 6); aviremic (n = 5)), and HIV-negative individuals (n = 7). Percentages were compared using the Mann-Whitney U test between the B-cell populations. Data shown are mean ±SEM. *p<0.05. PI, postinfection; ART, antiretroviral therapy.

Data were plotted in order to compare the percentage of IL-10 expressing cells between each B-cell population, within each patient group at various time points (Figure 3). Amongst the B-cell populations analyzed, the precursor MZ-like B-cells were the main expressors of IL-10 during the early phase of infection in both the rapid and classic progressor groups (Figure 3A, left panel; B, middle panel). This was also true for classic progressors in the chronic phase of infection (Figure 3B, right panel). All B-cell populations contributed equally to IL-10 expression in the slow progressor and HIV-negative donor groups (Figure 3C). Of note, plasma levels of IL-10 were found to be elevated throughout follow-up in all HIV-1-infected individuals, including ART-treated rapid progressors and aviremic slow progressors/EC (Figure S2C).

Overall, frequencies of total blood B-cells expressing IL-10 in all viremic HIV-1-infected individuals were significantly higher than those observed in HIV-negative donors. Amongst the B-cell populations analyzed, the relative percentage of IL-10 expressing cells is significantly increased within the precursor MZ-like, TI and memory populations. The most significant increase in cells expressing IL-10 was within the precursor MZ-like population. Importantly, we found unaltered percentages of total and of MZ-like B-cells expressing IL-10 in aviremic slow progressors/EC.

### Longitudinal monitoring of LT-α expression by blood B-cell populations in HIV-1-infected individuals with different rates of disease progression

**Figure 4:**
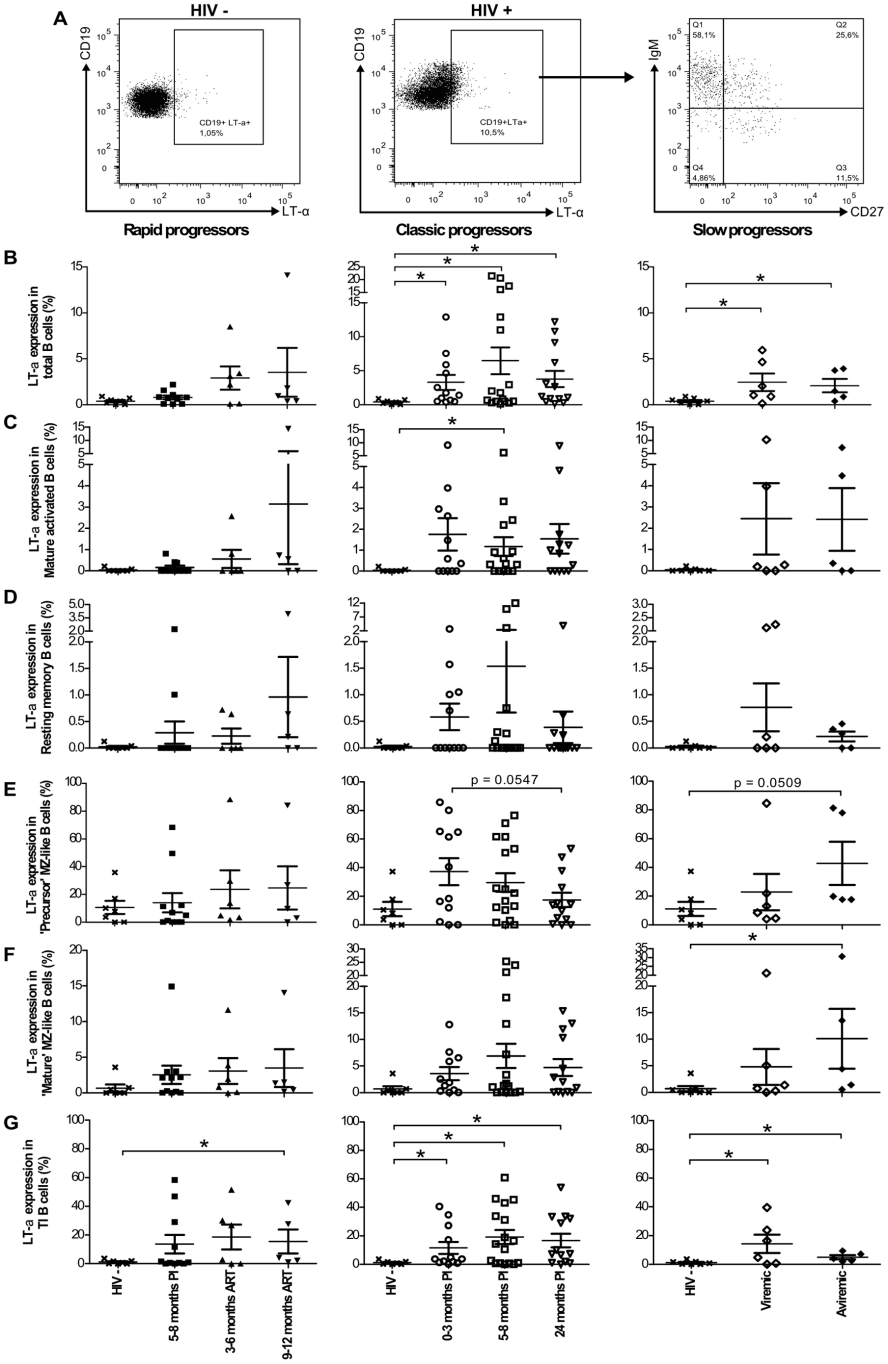
LT-α expression by blood B-cell populations. (A) Representative plot showing gating strategy on live total CD19+ B-cells from HIV- and HIV+ donors, expressing LT-α. Frequencies of cells expressing LT-α within (B) total, (C) mature activated, (D) resting switched memory, (E) precursor marginal zone (MZ)-like, (F) mature MZ-like and (G) transitional immature (TI) B-cells expressing LT-α in the blood of rapid progressors (left panels; 5–8 months PI (n = 11), 3–6 months ART (n = 6) and 9–12 months ART (n = 5)), classic progressors (middle panels; 0–3 months PI (n = 12), 5–8 months PI (n = 17), and 24 months PI (n = 13)), and viremic and aviremic slow progressors (EC) (right panels; viremic (n = 6); aviremic (n = 5)). The same values for HIV-negative donors in the left, middle and right panels are used as a control group (n = 7). Data are expressed as percentages of LT-α expression within each B-cell population. Cell population frequencies were compared using the Wilcoxon signed rank test and the Mann-Whitney U test for pairwise comparisons of different phases of infection within each group and between the study groups, respectively. Data shown are mean ±SEM. * p<0.05. PI, postinfection; ART, antiretroviral therapy.

Blood B-cell populations presented in Figure 1 were assessed *ex vivo* for intracellular LT-α based on the same strategy of flow-cytometry analysis depicted in Figure 1A. Figure 4A shows a representative profile of total live B-cells expressing LT-α in HIV- and HIV+ donors, and distribution within IgM and CD27 quadrants of expression. The data are presented as percentage of LT-α expressing cells within each B-cell population. There was a significant increase in frequencies of total B-cells expressing LT-α in the blood of both classic and slow progressors when compared to HIV-negative donors (Figure 4B). There was a trend for increased frequencies of total B-cells expressing LT-α following therapy in rapid progressors (Figure 4B, left panel). Similar patterns of LT-α expression were observed in mature activated, precursor MZ-like, mature MZ-like and TI B-cell populations (Figure 4C, E–G). The relative frequencies of resting switched memory B-cells expressing LT-α were not significantly affected (Figure 4D), as were those of naïve resting B-cells (data not shown). We did not detect changes in LT-α expression levels following analysis of geoMFI (data not shown). A recapitulative analysis of B-cell LT-α expression profiles is provided in Figure S4.

**Figure 5:**
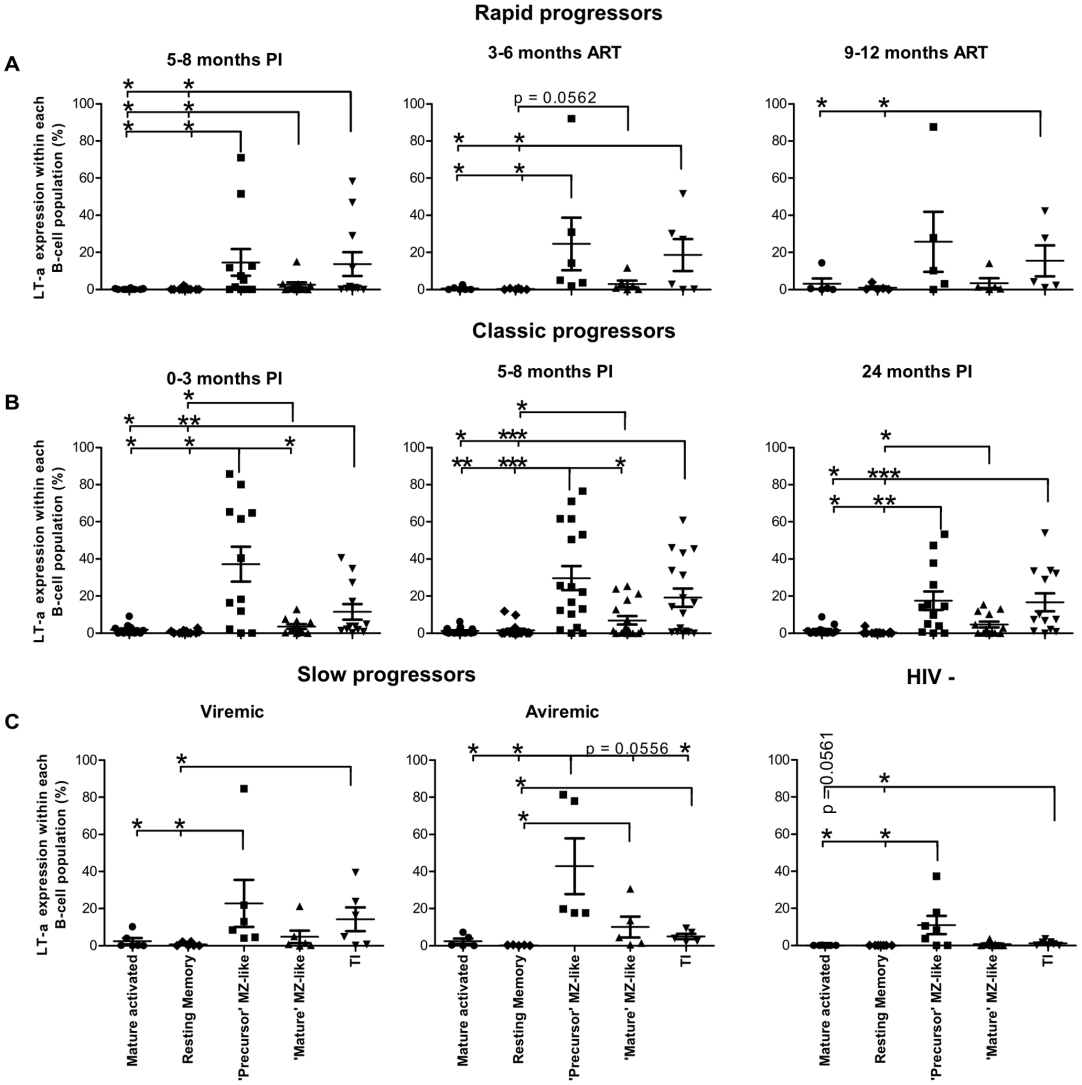
Percentage of LT-α expressing cells for each B-cell population, within each patient group. Percentage of LT-α expressing cells within mature activated, resting switched memory, precursor marginal zone (MZ)-like, mature MZ-like and transitional immature (TI) B-cells within (A) rapid progressors (5–8 months PI (n = 11), 3–6 months ART (n = 6) and 9–12 months ART (n = 5), (B) classic progressors (0–3 months PI (n = 12), 5–8 months PI (n = 17), and 24 months PI (n = 13)), (C) slow progressors (viremic (n = 6); aviremic (n = 5)), and HIV-negative individuals (n = 7). Percentages were compared using the Mann-Whitney U test between the B-cell populations. Data shown are mean ±SEM. * p<0.05, ** p<0.001, *** p<0.0001. PI, postinfection; ART, antiretroviral therapy.

Data were plotted in order to compare the percentage of LT-α expressing cells between each B-cell population, within each patient group at various time points (Figure 5). Amongst the B-cell populations analyzed, the precursor MZ-like and TI B-cells were the main contributors of LT-α expression during the acute, early and chronic phases of infection in all viremic HIV-1-infected individuals (Figure 5A-B; C, left panel). The precursor MZ-like B-cells were the main contributors of LT-α expression in the aviremic slow progressor/EC and HIV-negative groups (Figure 5C). Plasma levels of LT-α in all HIV-infected individuals were similar to those observed in HIV-negative donors (Figure S2D). Overall, increased relative frequencies of precursor MZ-like B-cells expressing LT-α appear associated with a better control of disease progression.

## Discussion

The percentages of total live blood CD19^+^ B-cells were lower in all HIV-1-infected individuals when compared to those observed in HIV-negative donors. However, despite the fact that precursor MZ-like B-cells may represent only a small fraction of circulating B-cells, their increased relative frequencies in the blood of HIV-1-infected rapid and classic progressors, may significantly influence the ability to mount efficient B-cell responses and disease progression. Indeed, given the frequent auto-reactive and cross-reactive repertoires of MZ type populations [12]–[13], the increased relative frequency of these cells may be associated with polyclonal B-cell activation and hyperglobulinemia previously reported for these individuals [14], leading to breakage of tolerance and autoimmune manifestations. Moreover, their increased relative proportions may reflect the host response to lymphopenia observed in rapid progressors [22] and the overall inflammation and high levels of BLyS/BAFF [23]–[29] previously found in the plasma and on surface of blood mDCs in these individuals [14]. In support of our findings, it has been shown that human blood and tonsil MZ-like B-cells naturally bind to gp120 through C-type lectins, and that this is increased by BLyS/BAFF *in vitro*, subsequently leading to polyclonal IgG and IgA responses, of which a fraction is gp120-specific [12]. Furthermore, the fact that the individuals presented herein experience microbial translocation [14] is in line with human MZ B-cells being particularly solicited in response to encapsulated bacteria [30]. In contrast, relative blood levels of precursor MZ-like B-cells in EC were similar to those observed in HIV-negative donors [14]. Moreover, EC had low-inflammatory profiles, unaltered levels of BLyS/BAFF and undetectable elements of microbial translocation [14], suggesting they are able to maintain a certain degree of immune integrity.

Interestingly, both viremic and aviremic slow progressors/EC had decreased relative frequencies of more mature MZ-like B-cells within their blood B-cell compartment when compared to both rapid and classic progressors, and HIV-negative donors. This finding could suggest that the capacity to recruit this population to peripheral sites may be beneficial to the “control” of disease progression. Intriguingly, a similar decrease in blood CD27^+^IgM^+^IgD^+^ MZ type B-cells has been recently reported in patients with MyD88, IRAK4, and TIRAP deficiencies [30]. Whether there is a relationship with the reduced frequencies of mature MZ-like B-cells we observe in slow progressors warrants further investigation.

Although our sample size was limited, we were able to accurately and reproducibly measure *ex vivo* IL-10 expression by blood B-cells. We found that in the context of detectable viremia, the percentages of total blood B-cells expressing IL-10 were increased when compared to HIV-negative individuals (Figure 2B). Amongst the B-cell populations analysed, the percentage of IL-10 expressing cells was significantly increased within the precursor MZ-like, TI and switched memory populations. Strikingly, the most significant increase in cells expressing IL-10 was within the precursor MZ-like population. Importantly, the relative increase in precursor MZ-like B-cells expressing IL-10 correlated with high levels of serum IgG1 and IgG3 in the early phase of HIV-1-infection in classic progressors (p = 0.0184; p = 0.0499, respectively), and with high levels of serum IgA in chronic classic progressors (p = 0.0479). These are likely to reflect polyclonal B-cell activation associated with the overall inflammatory condition we observed in HIV-1 progressors [14], [31], as B-cell IL-10 production has been shown to be modulated by BLyS/BAFF [32] and various signals, such as delivered via TLR, CD40 and the B-cell receptor (BCR) [16]–[17].

Our findings could also suggest an attempt from the host to dampen the overwhelming inflammatory burden by soliciting regulatory functions from various B-cell populations, such as switched memory, TI and MZ-like B-cells, which have been variably associated with Breg capacity [16]–[17]. Of course, we cannot rule out that precursor MZ-like B-cells are more sensitive to the overall HIV disease process, but it may be that they represent a population more prone to Breg potential. Frequencies of populations with similar characteristics and Breg attributes have been shown to be increased in the blood of systemic lupus erythematosus (SLE) and rheumatoid arthritis (RA) patients, albeit these cells were refractory to *in vitro* stimulation, produced less IL-10 and lacked suppressive activity [16]. Likewise, it is possible that in the context of HIV disease progression, populations ascribed to Breg potential may have exhausted and/or dysregulated regulatory capacities.

Nevertheless, the overall outcome of increased frequencies of B-cells expressing IL-10 may well be to sustain chronic B-cell activation and dysregulation, and may lead to imbalanced Treg/Teffector ratios [15] associated with HIV disease progression [33]. Furthermore, high level of Breg activity was recently shown to be involved in suppression of antiviral T effector functions *in vitro* during HIV [18] and chronic hepatitis B [34] infections, thus impending viral eradication.

In the context of HIV disease control, such as encountered in EC, the frequencies of total and MZ-like B-cells expressing IL-10, remained unaltered. Although increased frequencies of resting switched memory and TI blood B-cells expressing IL-10 were found in EC, these were lower than in the MZ-like populations. These findings suggest that EC are capable, to a certain extent, to regulate their IL-10 expression status and possibly “Breg” activity, and this may help in maintaining homeostasis of immune responses associated with HIV control.

Although high levels of LT-α have been associated with autoimmune and inflammatory conditions [19], plasma levels of LT-α in all HIV-1-infected subjects were similar to those found in HIV-negative donors. Amongst the B-cell populations analyzed, the precursor MZ-like B-cells were the main contributors to LT-α expression in all subjects regardless of HIV-1 infection and rate of disease progression. The percentages of IL-10 expressing cells were significantly greater than those of LT-α within most B-cell populations except for precursor MZ-like and TI B-cells, which frequencies of IL-10 and LT-α expression were similar. Importantly, EC had increased frequencies of MZ-like B-cells expressing LT-α resulting in higher LT-α to IL-10 expression ratios. Studies in the murine system have elegantly shown the importance of LT-α in the organization and maintenance of lymphoid structures, as well as in the modulation of immune responses [19], through a process involving a positive CXCL13 feedback loop [35]. Interestingly, plasma levels of CXCL13 were higher in viremic individuals compared to HIV-negative donors [36] and EC (Chagnon-Choquet J unpublished data), suggesting that the higher relative percentages of MZ-like B-cells expressing LT-α in EC may help to maintain CXCL13 levels and a certain lymphoid homeostasis.

The first-line MZ-like B-cells described herein likely contribute directly to the generation of HIV-specific Abs. Indeed, human MZ B-cells express IGHV1-2*02 [37], which has been repeatedly found to encode mutated HIV broadly neutralizing Abs (bNAbs), such as VRC01 [38]. Interestingly, the recent characterization of transient Gp41-specific IgA in mucosal genital fluids from patients within the first weeks after HIV transmission, suggest these Abs might have originated from first-line B-cell populations [39]. Of note, BLyS/BAFF was elevated immediately preceding the appearance of these Abs. Interestingly, repeated treatment of mice with BLyS/BAFF increased the MZ compartment, and generated an increased response to Env immunization and bNAbs from these animals [40]. Understanding the dynamics of BLyS/BAFF and its role in homeostasis of immune responsiveness appears pivotal to the design of vaccine strategies soliciting protective B-cell responses. Based on our observations, the capacity to contain BLyS/BAFF expression levels seems associated with control of disease progression, whereas excessive BLyS/BAFF may promote immune dysregulation and disease progression [41].

The findings reported herein are in line with growing evidence suggesting that first-line B-cell responses are involved in the battle against HIV [42], and with the importance of MZ type B-cells in health and disease [12]-[13]. Although further characterization is required to identify the exact nature of the MZ-like B-cells described herein, they certainly deserve further interest.

## Supporting Information

Figure S1 **Flow-cytometry gating strategy based on fluorescence minus one and isotype controls.** Dot plots showing gating strategy based on fluorescence minus one and isotype control for (A) CD19, (B) CD21, (C) CD27 and IgM, (D) CD1c and CD10, (E) IL-10 and (F) LT-α.
(TIF)

Figure S2 **Longitudinal variations of blood CD4+ T-cell counts, viral loads and IL-10 and LT-α concentrations of HIV-1 infected individuals.** (A) Blood CD4+ T-cell counts (cell/mm3) were determined by flow-cytometry in rapid progressors (left panel; 0–3 months PI (n = 13), 5–8 months PI (n = 13), 3–6 months ART (n = 7), 9–12 months ART(n = 5)), classic progressors (middle panel; 0–3 months PI (n = 16), 5–8 months PI (n = 16), 24 months PI (n = 11)), and slow progressors (right panel; viremic (n = 6), aviremic (n = 5)). (B) Viral loads (log copies/ml) were quantified by *in vitro* signal amplification nucleic acid probe assay of HIV-1 RNA (bDNA) in the plasma of rapid progressors (left panel; 0–3 months PI (n = 13), 5–8 months PI (n = 13), 3–6 months ART (n = 7), 9–12 months ART (n = 6)), classic progressors (middle panel; 0–3 months PI (n = 17), 5–8 months PI (n = 17), 24 months PI (n = 11)), and slow progressors (right panel; viremic (n = 6), aviremic (n = 6)). (C) Concentrations of IL-10 measured longitudinally in the plasma of rapid progressors (left panel; 0–3 months PI (n = 12), 5–8 months PI (n = 13), 3–6 months ART (n = 9), 9–12 months ART (n = 7)), classic progressors (middle panel; 0–3 months PI (n = 17), 5–8 months PI (n = 17), 24 months PI (n = 11)) and slow progressors (right panel; viremic (n = 7), aviremic (n = 5)). The same values for HIV-negative donors (n = 20) in the left, middle and right panels are used as a control group. (D) Concentrations of LT-α measured longitudinally in the plasma of rapid progressors (left panel; 0–3 months PI (n = 10), 5–8 months PI (n = 12), 3–6 months ART (n = 6), 9–12 months ART (n = 4)), classic progressors (middle panel; 0–3 months PI (n = 14), 5–8 months PI (n = 10), 24 months PI (n = 9)) and slow progressors (right panel; viremic (n = 4), aviremic (n = 4)). The same values for HIV-negative donors (n = 18) in the left, middle and right panels are used as a control group. Cell populations, viral loads and plasma concentrations were compared using the Wilcoxon signed-rank test and Mann-Whitney U test for pairwise comparisons of different phases of infection within each group and between the study groups, respectively. Data shown are mean ±SEM. Significance levels are shown as * p<0.05, ** p<0.001, *** p<0.0001. PI, post-infection; ART, antiretroviral therapy.
(TIF)

Figure S3 **Contribution of each blood B-cell population to IL-10 expression.** Percentages of IL-10 expression within each B-cell population; ‘mature’ marginal zone (MZ)-like (purple), ‘precursor’ MZ-like (cherry red), mature activated (yellow), transitional immature (TI) (blue) and resting switched memory (orange) B-cells, for rapid progressors (left panel; 5–8 months PI (n = 11), 3–6 months ART (n = 6), 9–12 months ART (n = 5)), classic progressors (middle panel; 0–3 months PI (n = 12), 5–8 months PI (n = 17), 24 months PI (n = 13)), and slow progressors (right panel; viremic (n = 6), aviremic (n = 5)). The same value for HIV-negative donors in the left, middle and right panels are used as a control group (n = 7). Cell population frequencies were compared using the Mann-Whitney U test between the study groups. Data shown are mean ± SEM. * p<0.05. PI, post-infection; ART, antiretroviral therapy.
(TIF)

Figure S4 **Contribution of each blood B-cell population to LT-α expression.** Percentages of LT-α expression within each B-cell population; ‘mature’ marginal zone (MZ)-like (purple), ‘precursor’ MZ-like (cherry red), mature activated (yellow), transitional immature (TI) (blue) and resting switched memory (orange) B-cells, for rapid progressors (left panel; 5–8 months PI (n = 11), 3–6 months ART (n = 6), 9–12 months ART (n = 5)), classic progressors (middle panel; 0–3 months PI (n = 12), 5–8 months PI (n = 17), 24 months PI (n = 13)), and slow progressors (right panel; viremic (n = 6), aviremic (n = 5)). The same value for HIV-negative donors in the left, middle and right panels are used as a control group (n = 7). Cell population frequencies were compared using the Mann-Whitney U test between the study groups. Data shown are mean ±SEM. * p<0.05. PI, post-infection; ART, antiretroviral therapy.
(TIF)
