# Supplementary figures and images for: IL-10 and Lymphotoxin-α Expression Profiles within Marginal Zone-Like B-Cell Populations Are Associated with Control of HIV-1 Disease Progression
Source: PLoS One. 2014 Jul 8;9(7):e101949. doi: 10.1371/journal.pone.0101949 (PMC4087016; doi:10.1371/journal.pone.0101949)

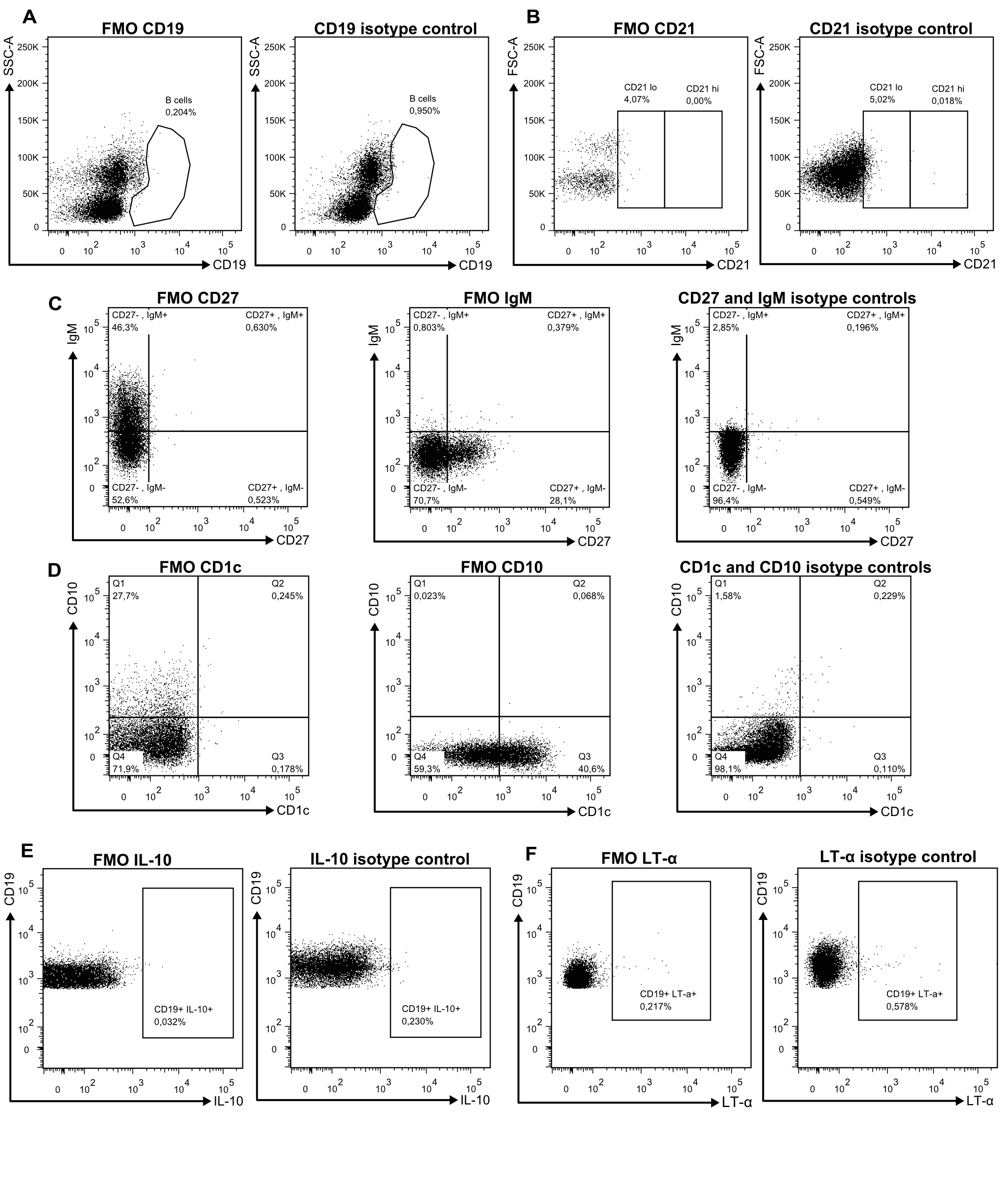

Supplement: Figure S1 — Flow-cytometry gating strategy based on fluorescence minus one and isotype controls. Dot plots showing gating strategy based on fluorescence minus one and isotype control for (A) CD19, (B) CD21, (C) CD27 and IgM, (D) CD1c and CD10, (E) IL-10 and (F) LT-α. (TIF) [file pone.0101949.s001.tif]

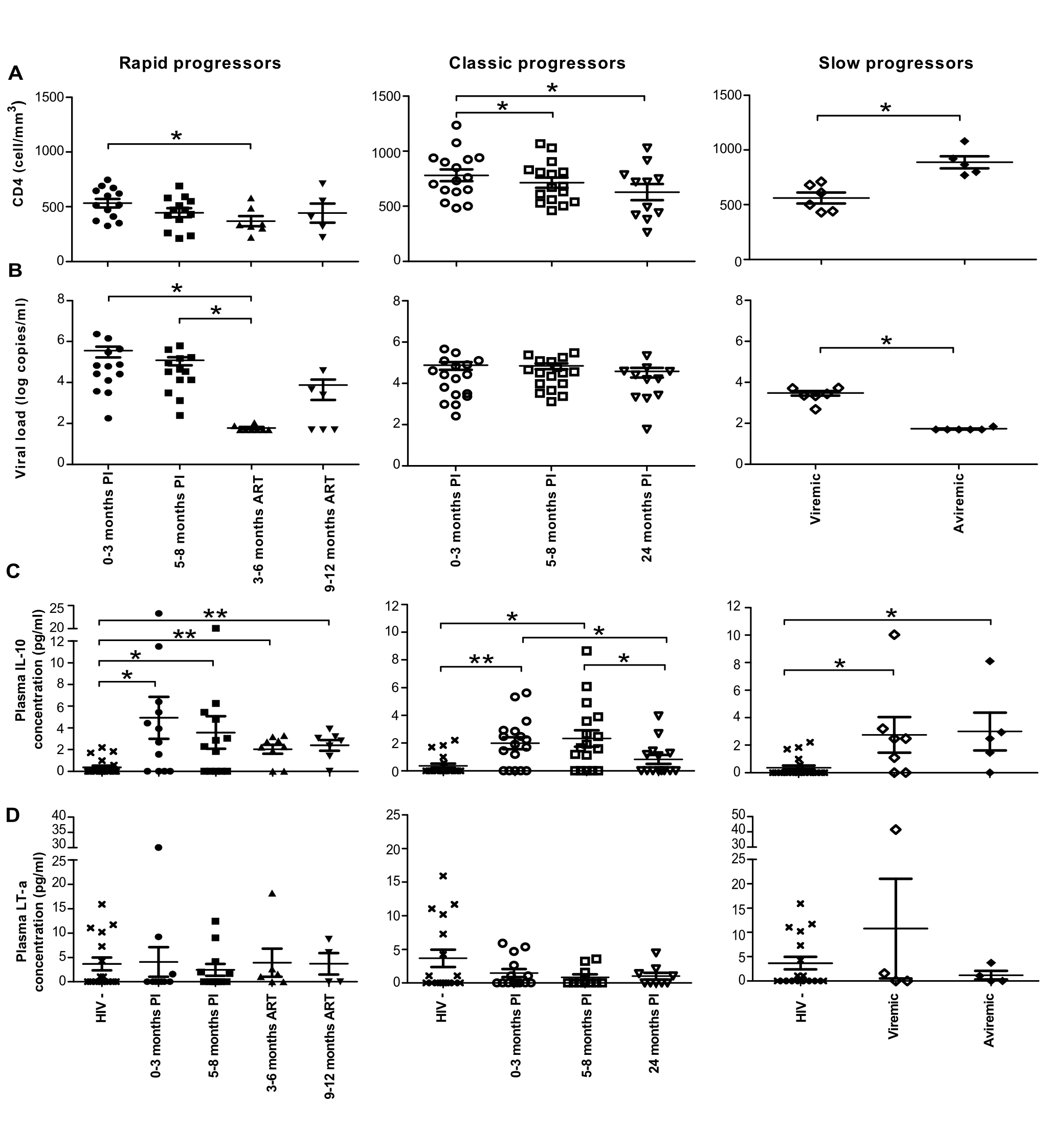

Supplement: Figure S2 — Longitudinal variations of blood CD4+ T-cell counts, viral loads and IL-10 and LT-α concentrations of HIV-1 infected individuals. (A) Blood CD4+ T-cell counts (cell/mm3) were determined by flow-cytometry in rapid progressors (left panel; 0–3 months PI (n = 13), 5–8 months PI (n = 13), 3–6 months ART (n = 7), 9–12 months ART(n = 5)), classic progressors (middle panel; 0–3 months PI (n = 16), 5–8 months PI (n = 16), 24 months PI (n = 11)), and slow progressors (right panel; viremic (n = 6), aviremic (n = 5)). (B) Viral loads (log copies/ml) were quantified by in vitro signal amplification nucleic acid probe assay of HIV-1 RNA (bDNA) in the plasma of rapid progressors (left panel; 0–3 months PI (n = 13), 5–8 months PI (n = 13), 3–6 months ART (n = 7), 9–12 months ART (n = 6)), classic progressors (middle panel; 0–3 months PI (n = 17), 5–8 months PI (n = 17), 24 months PI (n = 11)), and slow progressors (right panel; viremic (n = 6), aviremic (n = 6)). (C) Concentrations of IL-10 measured longitudinally in the plasma of rapid progressors (left panel; 0–3 months PI (n = 12), 5–8 months PI (n = 13), 3–6 months ART (n = 9), 9–12 months ART (n = 7)), classic progressors (middle panel; 0–3 months PI (n = 17), 5–8 months PI (n = 17), 24 months PI (n = 11)) and slow progressors (right panel; viremic (n = 7), aviremic (n = 5)). The same values for HIV-negative donors (n = 20) in the left, middle and right panels are used as a control group. (D) Concentrations of LT-α measured longitudinally in the plasma of rapid progressors (left panel; 0–3 months PI (n = 10), 5–8 months PI (n = 12), 3–6 months ART (n = 6), 9–12 months ART (n = 4)), classic progressors (middle panel; 0–3 months PI (n = 14), 5–8 months PI (n = 10), 24 months PI (n = 9)) and slow progressors (right panel; viremic (n = 4), aviremic (n = 4)). The same values for HIV-negative donors (n = 18) in the left, middle and right panels are used as a control group. Cell populations, viral loads and plasma concentrations wer [file pone.0101949.s002.tif]

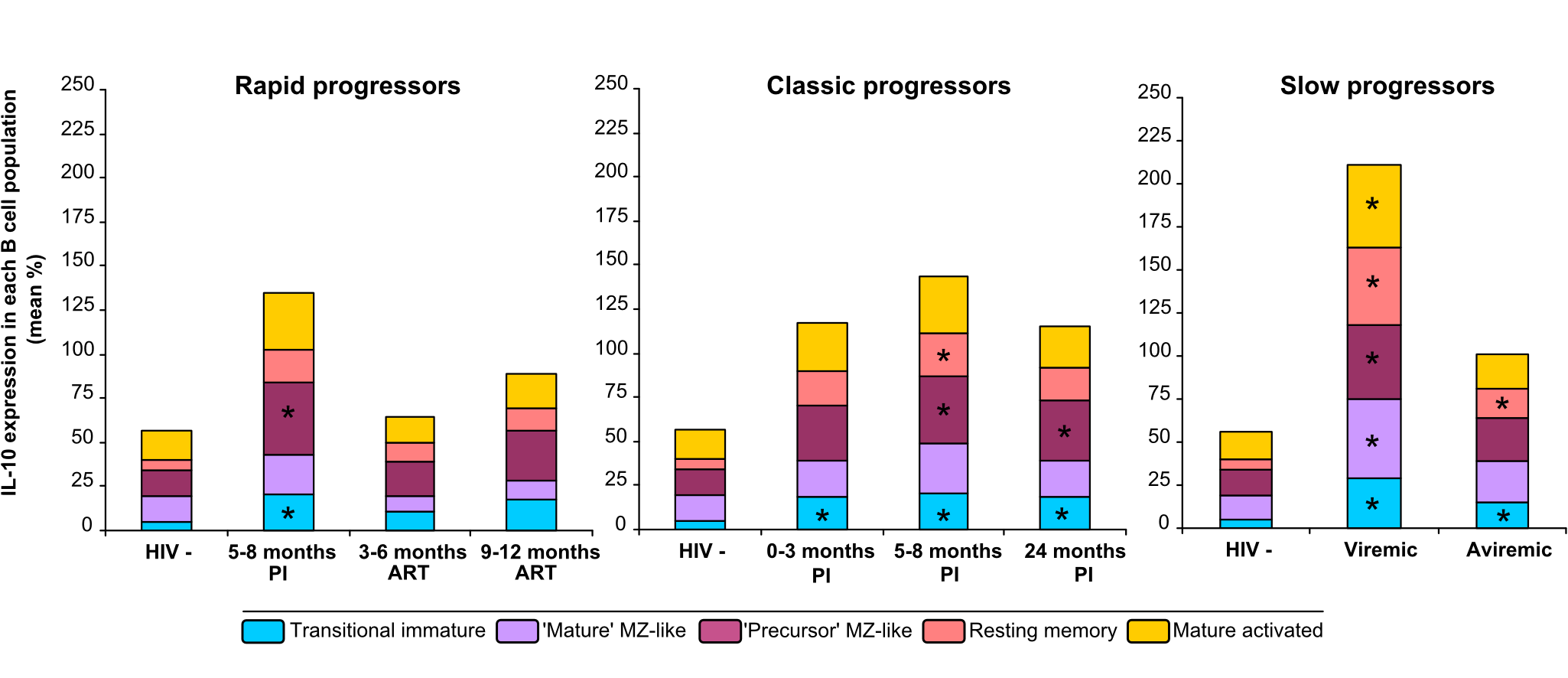

Supplement: Figure S3 — Contribution of each blood B-cell population to IL-10 expression. Percentages of IL-10 expression within each B-cell population; ‘mature’ marginal zone (MZ)-like (purple), ‘precursor’ MZ-like (cherry red), mature activated (yellow), transitional immature (TI) (blue) and resting switched memory (orange) B-cells, for rapid progressors (left panel; 5–8 months PI (n = 11), 3–6 months ART (n = 6), 9–12 months ART (n = 5)), classic progressors (middle panel; 0–3 months PI (n = 12), 5–8 months PI (n = 17), 24 months PI (n = 13)), and slow progressors (right panel; viremic (n = 6), aviremic (n = 5)). The same value for HIV-negative donors in the left, middle and right panels are used as a control group (n = 7). Cell population frequencies were compared using the Mann-Whitney U test between the study groups. Data shown are mean ± SEM. * p<0.05. PI, post-infection; ART, antiretroviral therapy. (TIF) [file pone.0101949.s003.tif]

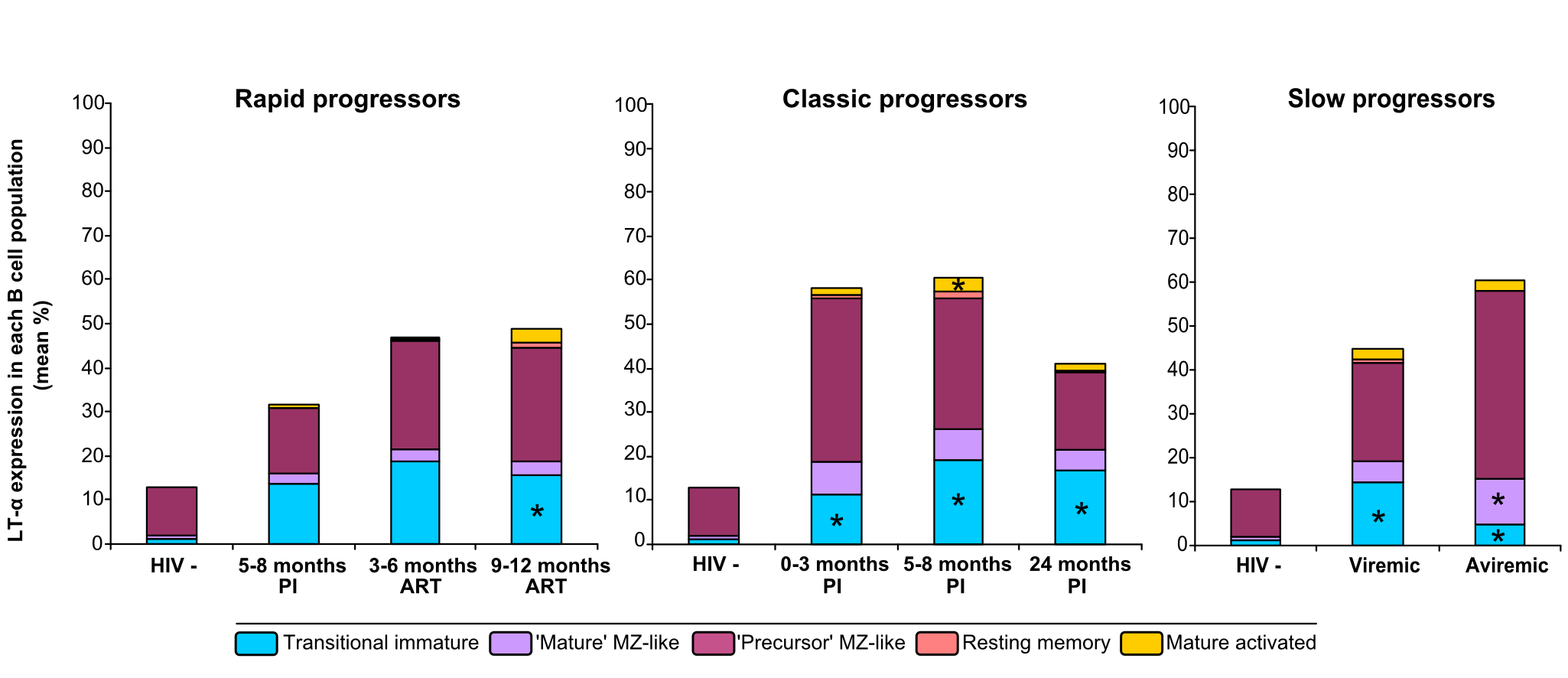

Supplement: Figure S4 — Contribution of each blood B-cell population to LT-α expression. Percentages of LT-α expression within each B-cell population; ‘mature’ marginal zone (MZ)-like (purple), ‘precursor’ MZ-like (cherry red), mature activated (yellow), transitional immature (TI) (blue) and resting switched memory (orange) B-cells, for rapid progressors (left panel; 5–8 months PI (n = 11), 3–6 months ART (n = 6), 9–12 months ART (n = 5)), classic progressors (middle panel; 0–3 months PI (n = 12), 5–8 months PI (n = 17), 24 months PI (n = 13)), and slow progressors (right panel; viremic (n = 6), aviremic (n = 5)). The same value for HIV-negative donors in the left, middle and right panels are used as a control group (n = 7). Cell population frequencies were compared using the Mann-Whitney U test between the study groups. Data shown are mean ±SEM. * p<0.05. PI, post-infection; ART, antiretroviral therapy. (TIF) [file pone.0101949.s004.tif]
